#  The metabolome profiling of obese and non-obese individuals: Metabolically healthy obese and unhealthy non-obese paradox

**DOI:** 10.22038/IJBMS.2019.37885.9004

**Published:** 2020-02

**Authors:** Saeed Chashmniam, Nahid Hashemi Madani, BiBi Fatemeh Nobakht Mothlagh Ghoochani, Nahid Safari-Alighiarloo, Mohammad E. Khamseh

**Affiliations:** 1 Department of Chemistry, Sharif University of Technology, Tehran, Iran; 2 Endocrine Research Center, Institute of Endocrinology and Metabolism, Iran University of Medical Sciences, Tehran, Iran; 3 Chemical Injuries Research Center, Systems Biology and Poisoning Institute, Baqiyatallah University of Medical Sciences, Tehran, Iran

**Keywords:** Metabolically healthy obese, Metabolically unhealthy- non-obese, Metabolomics, Obesity, ^ 1^H-NMR

## Abstract

**Objective(s):**

The molecular basis of “metabolically healthy obese” and “metabolically unhealthy non-obese” phenotypes is not fully understood. Our objective was to identify metabolite patterns differing in obese (metabolically healthy vs unhealthy (MHO vs MUHO)) and non-obese (metabolically healthy vs unhealthy (MHNO vs MUHNO)) individuals.

**Materials and Methods:**

This case-control study was performed on 86 subjects stratified into four groups using anthropometric and clinical measurements: MHO (21), MUHO (21), MHNO (22), and MUHNO (22). Serum metabolites were profiled using nuclear magnetic resonance (NMR). Multivariate analysis was applied to uncover discriminant metabolites, and enrichment analysis was performed to identify underlying pathways.

**Results:**

Significantly higher levels of glutamine, asparagine, alanine, L-glutathione reduced, 2-aminobutyrate, taurine, betaine, and choline, and lower level of D-sphingosine were observed in MHO group compared with MUHO. In comparison of MHNO and MUHNO groups, significantly lower levels of alanine, glycine, glutamine, histidine, L-glutathione reduced, and betaine, and higher levels of isoleucine, L-proline, cholic acid, and carnitine appeared in MUHNO individuals. Moreover, significantly affected pathways included amino acid metabolism, urea cycle and ammonia recycling in MUHO subjects and glutathione metabolism, amino acid metabolism, and ammonia recycling in MUHNO members.

**Conclusion:**

Literature review helped us to hint that altered levels of most metabolites might associate to insulin sensitivity and insulin resistance in MHO and MUHNO individuals, respectively. Besides, abnormal amino acid metabolism and ammonia recycling involved in unhealthy phenotypes (MUHO, MUHNO) might be associated with insulin resistance.

## Introduction

Obese individuals are at increased risk of developing a wide range of diseases, especially cardiovascular disease and type 2 diabetes mellitus (1). However, not all obese individuals will develop obesity-related diseases. According to previous studies, the metabolically healthy obesity (MHO) group may be protected from metabolic diseases compared with the metabolically unhealthy obesity (MUHO) one (2, 3). Interestingly, it has been reported that a subgroup of non-obese individuals has a high risk of cardiometabolic complications (4). This distinct subset is considered as metabolically unhealthy (MUHNO) compared with metabolically healthy non-obese (MHNO).

It has been estimated that 13% to 29% of obese individuals represent the MHO phenotype, depending on the population studied. They bear a relatively healthy metabolic status such as normal insulin sensitivity, blood lipid profile, and fasting glucose level (5). However, the evidence proposes that despite “healthy” metabolic profiles, these individuals may still be at increased risk for adverse long term outcomes (6). Although the underlying mechanisms of the metabolic regulation are not yet clear, several studies have uncovered relevant factors for distinguishing the MHO and MUHO phenotypes. For instance, a higher level of adiponectin, lower visceral fat content, a significantly lower percentage of ectopic fat, especially in the muscles and liver, have been reported in MHO individuals copmpared with MUHO (7-9). In parallel, numerous studies suggest that the prevalence of metabolically unhealthy normal-weight is about 10% to 37% depending on the explored ethnic population (5). The unhealthy normal-weight individuals possess cardiometabolic abnormalities like reduced insulin sensitivity and dyslipidemia (10). The result of a study indicated that in unhealthy normal-weight subjects, metabolic risks such as elevated glucose, dyslipidemia, and hypertension are more strongly associated with a relatively low leg fat mass than with high subcutaneous abdominal fat mass, visceral obesity, or fatty liver (11). Therefore, it appears substantive to elucidate the metabolic and molecular basis underlying the metabolically normal and abnormal obese and non-obese individuals more, as this may lead to more appropriate health care strategies.

According to evidence, metabolite profiling (i.e., metabolomics) is an appropriate approach that could uncover differences in metabolism between each group of individuals (12-14). Since small molecule metabolites introduce organisms’ conditions at the given moment, they represent the dynamic physiological situation in response to pathological exposures or genetic modifications (15). Nuclear magnetic resonance (NMR) spectroscopy is a rapid, non-destructive, and high-throughput analytical method with the capability of simultaneous detection, identification, and quantification of several hundreds of metabolites in the biological matrix (16, 17). Several studies compared metabolite profiles in individuals characterized as metabolically healthy *vs* unhealthy and reported a cluster of metabolites distinguishing obesity phenotypes. Overall, they suggested that amino acids, phospholipids, and acyl-carnitine significantly differed between MHO and MUHO groups (15, 18, 19). Although previous metabolomic studies have been used to identify the metabolome fingerprints in obesity to investigate the complex molecular differences between the MHO and MUHO subjects (15, 18, 19), this study revealed metabolome profiling in younger obese adults compared with others. Besides, to the best of our knowledge, the metabolome profiles of MHNO vs MUHNO individuals have not yet been investigated.

In the present study, we investigated the metabolite patterns of obese (MHO *vs* MUHO) and non-obese (MHNO *vs* MUHNO) individuals to distinguish a perturbed metabolic state from normal ones. To achieve this objective, we performed a metabolomics study of similar age and BMI as well as approximately sex paired samples in each group. Serum metabolites were initially profiled using untargeted ^1^H-NMR spectroscopy coupled with multivariate statistical analysis (principal component analysis (PCA) and orthogonal partial least squares discriminant analysis (OPLS-DA)). We also mapped relevant metabolites to their corresponding metabolic pathways to profile the underlying mechanisms of metabolic regulation among obese and non-obese individuals. 

## Materials and Methods

***Study subjects***

This case-control study was performed on 86 subjects stratified into four groups according to the BMI and health status: MHO (n=21), MUHO (n=21), MHNO (n=22), and MUHNO (n=22). They were randomly (simple randomization) recruited from a health screening program carried out on a population of post-graduate students at the Institute of Endocrinology and Metabolism, Iran University of Medical Sciences, Tehran, Iran. Participants were individually matched in groups with similar age and BMI as well as approximately for sex to minimize potential confounding effects. The exclusion criteria were as follows: Cigarette smoking, heavy alcohol drinking, being diagnosed as diabetes mellitus, any known inflammatory diseases, or cancer. Obesity was defined as body mass index (BMI) ≥30 kg/m2 and participants with BMI<30 kg/m2 were considered as being non-obese. The protocol was approved by the medical ethical committee of Iran University of Medical Sciences, according to the Declaration of Helsinki. Written informed consent was obtained from all study participants.

Metabolically healthy subjects needed to meet all the following criteria: systolic blood pressure (SBP) <140 mmHg and diastolic blood pressure (DBP) <90 mmHg, no antihypertensive drug use, fasting blood sugar (FBS) <126 mg/dl, no hypoglycemic agents use, and serum levels of high-density lipoprotein cholesterol (HDL-C) ≥40 mg/dl in men or ≥50 mg/dl in women. The absence of any of the above criteria put the participant in the metabolically unhealthy status (20). Insulin resistance was defined as Homeostatic Model Assessment (HOMA-IR) index of ≥2.5 calculated using this formula: fasting insulin in µIU/ml* fasting blood sugar in mg/dl /405. 

***Anthropometric and laboratory measurements***

Anthropometrical measurements were performed prior to blood sampling. Bodyweight and height were measured using standard methods by trained staff. BMI was calculated as body weight (kg) / body height (m2). Blood pressure including systolic SBP and DBP were measured twice with Omron mercury sphygmomanometers for each of the participants while they were seated, after 5 min of rest, and the average of two measurements was calculated. Venous blood samples were obtained in the morning after an overnight fast. Biochemical indexes including FBS, triglyceride (TG), and HDL-C were measured with an automated instrument (Liasys autoanalyzer) using Pars Azmun kits. Serum insulin level was determined by applying a chemiluminescence immunoassay (ECLIA) Roche Kit on an Elecsys 2010 analyzer (Hitachi High-Technologies Corporation, Japan).

***Serum metabolites measurements***

Fasting blood samples were collected from the subjects in the morning and centrifuged at 2500 rpm for 10 min at room temperature. The serum samples were stored at −80 ^°^C until further analysis. At the time of NMR measurement, 600 μl of serum samples were added to 60 μl D2O and then transferred into a 5 mm NMR tube for NMR analysis. All serum samples were analyzed using one-dimensional ^1^H-NMR spectra with a 500-MHz Bruker DRX spectrometer, operating at 500.13 MHz. It was equipped with 5 mm high-quality NMR tubes (Sigma Aldrich,Johannesburg, RSA), with the temperature set at 298 K. To facilitate the detection of low molecular-weight species, the Carr-Purcell-Meiboom-Gill (CPMG) spin-echo pulse sequence, π/2-tD-π-tD, were performed for all one dimensional spectra. Acquisition parameters were spectral width 8389.26 Hz, time-domain points 32 K, relaxation delay 2 sec, number of scans 154, and acquisition time 1.95 sec, spectrum size 32 K, and line broadening 0.3 Hz (21). All of the ^1^H-NMR spectra were manually corrected for phase and baseline distortion using XWINNMR (version3.5, Bruker Spectrospin Ltd, Billerica, MA). All spectra were referenced to the methyl group of lactate at 1.336 ppm and D2O (NMR solvent). To exploit all metabolic information embedded in the spectra, each NMR spectrum was segmented into 408 regions of equal width (typically 0.02 ppm) using the ProMetab software (ver. 3.3) in MATLAB (version 6.5.1, The MathWorks, Cambridge, UK) (22). Unwanted spectral regions (4.2–5.2 ppm) corresponding to the residual of water were removed. An integral value was calculated for each segmented region and log-transformed to normalize for each sample, mean-centered to facilitate model interpretation, and unit variance scaled to consider high and low range variables with equal importance prior to the multivariate analysis.

***Statistical analysis***

The final data set was imported to SIMCA version 14.0 (Umetrics, Umea, Sweden) for multivariate statistical analysis. The Hotelling’s T2 region defined the 95% confidence interval of the modeled variation. Unsupervised principal component analysis (PCA) was applied on the dataset to separate groups and recognize possible outliers. A supervised orthogonal partial least-squares discriminant analysis (OPLS-DA) was used to construct predictive models and identify metabolite fingerprint differences. The model was assessed using the cross-validation test by considering three parameters (R2X, R2Y, and Q2). The classification performance (sensitivity and specificity) of OPLS-DA models and the area under the curve (AUC) of ROC were calculated by seven-fold cross-validation using SPSS software (Version 23.0, SPSS, Inc.). Variable influence on projection (VIP) was used to rank the overall contribution of each metabolite to the OPLS-DA model. Besides, two-tailed t-test was used to detect differential metabolites between groups using the SPSS software package, and a *P*-value of less than 0.05 was considered to be statistically significant. Fold change was calculated, and the most important metabolite candidates were selected on the basis of fold change >1.5. Correlation analysis was performed for each significant metabolite with HOMA-IR. 

## Results

***Participant’s characteristics***

The general characteristics of the 86 participants are shown in Table 1. Participants were stratified based on BMI and health status into four groups: 21 MHO *vs* 21 MUHO individuals and 22 MHNO vs 22 MUHNO counterparts. Each of the paired groups (obese and non-obese groups) was similar in age and BMI as well as approximately for sex variables as expected by matching design. There were no significant differences for SBP, DBP, FBS, and TG between paired groups. Total cholesterol was significantly higher in MHNO vs MUHNO (176 vs 157 mg/dl; *P*<0.003). HDL was significantly higher in MHO compared with MUHO (46 vs 35 mg/dl; *P*<0.00) and in MHNO compared with MUHNO (45.5 vs 35.5 mg/dl; *P*<0.00). Insulin level and HOMA-IR were significantly higher in MUHO compared with MHO (14.26 vs 11.48 µIU/L; *P*<0.04) and (2.92 vs 2.49; *P*<0.04), as well as in MUHNO compared with MHNO (9.93 vs 7.78 µIU/L; *P*<0.01) and (2.1 vs 1.6; *P*<0.02), respectively. 

***Serum metabolites in obese and non-obese groups***

**Figure 1:**
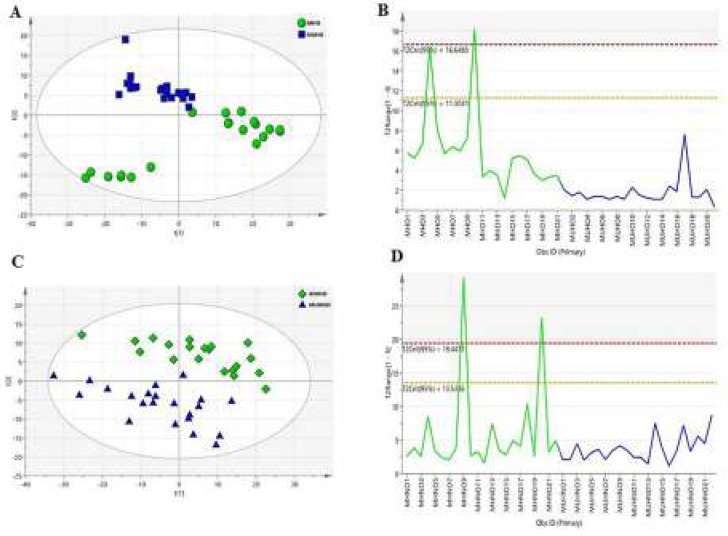
Multivariate statistical analysis to visualize the separation of groups and determination of outliers. PCA score plots with all variables unit variance scaled for comparison between (A) metabolically healthy obese (MHO) vs metabolically unhealthy obese (MUHO), (C) in metabolically healthy non-obese (MHNO) vs metabolically unhealthy non-obese (MUHNO) groups, and 0.95 Hotelling’s T2 test for outlier detection, (B) metabolically healthy obese (MHO) vs metabolically unhealthy obese (MUHO), (D) in metabolically healthy non-obese (MHNO) vs metabolically unhealthy non-obese (MUHNO) groups

**Figure 2:**
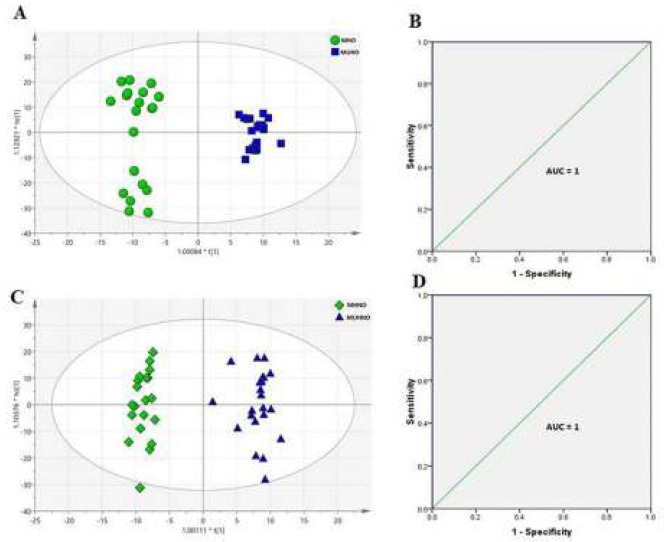
Predictive model constructed with supervised OPLS-DA method and validation of models. (A) OPLS-DA score plot for the comparison of metabolically healthy obese (MHO) vs metabolically unhealthy obese (MUHO), (C) OPLS-DA score plot for the comparison of metabolically healthy non-obese (MHNO) vs metabolically unhealthy non-obese (MUHNO) subjects, (B, D) ROC curves for evaluating the predictive models of OPLS-DA

Untargeted metabolite analysis of serums from obese and non-obese individuals was performed using the ^1^H-NMR approach. Multivariate data analyses were further used to obtain the most differential metabolites between individuals of the two groups. A fraction of the dataset was used for further multivariate analysis, containing top abundance of bins that filtered against the minor values. This fraction of data was found to be representative of most differences between the groups. The PCA score plots were used with two principal components (PC1 and PC2). PC1 plots for both groups are represented in Figure S1, and PC2 plots provided with R2X (cum)=0.827 and Q2 (cum)=0.75 and R2X (cum)=0.894 and Q2 (cum)=0.773 for obese and non-obese groups, respectively, (Figures 1A, 1C). To uncover observations lying outside, the 0.95 Hotelling’s T2 ellipse was also performed. There were four outliers for each comparison (MHO4, MHO10, MHNO9, and MHNO20) (Figures 1B, D, and Figure S1). OPLS-DA classification model was established to gain the metabolic differences between the two groups (MHO vs MUHO) and (MHNO vs MUHNO), respectively. The OPLS-DA score plot displayed a good separation R2X (cum)=0.768, R2Y (cum)=0.969, and Q2 (cum)=0.945 between MHO and MUHO groups (Figure 2A). According to the score plot of the OPLS-DA model, MHNO and MUHNO individuals were discriminated obviously with R2X (cum)=0.63, R2Y (cum)=0.961, and Q2 (cum)=0.919 (Figure 2C). Moreover, ROC curves were plotted based on the predicted response values in both OPLS-DA models (Figures 2B, 2D). The AUC for the ROC curves was 1 for both OPLS-D models. S-plots were also depicted for each of the comparisons, and more significant ppms were inserted on them (Figure S2). 

**Figure 3:**
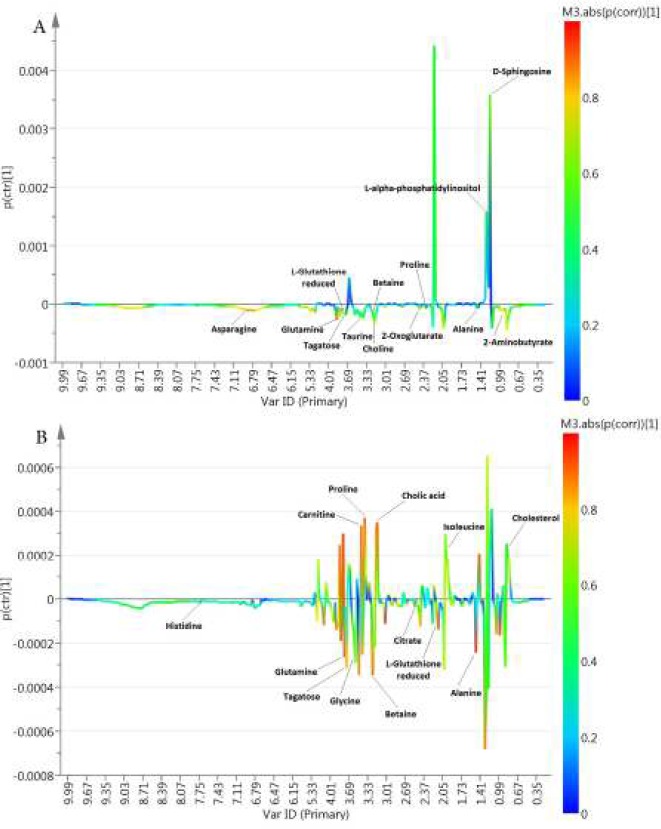
S-line plots obtained for (A) the comparison of metabolically healthy obese (MHO) vs metabolically unhealthy obese (MUHO) and (B) the comparison of metabolically healthy non-obese (MHNO) vs metabolically unhealthy non-obese (MUHNO) subjects

Consequently, it revealed that the NMR-based fingerprints have the potential to be used to distinguish the two groups (MHO vs MUHO) and (MHNO vs MUHNO). Metabolites exhibiting significant changes (*P*<0.05) and fold change >1.5, which were responsible for the discrimination between (MHO vs MUHO) and (MHNO vs MUHNO) individuals, were listed in Tables 2 and 3. Besides, S-line plots of each comparison were also displayed, and the differential metabolites were shown on them (Figure 3). There were significantly higher levels of alanine, glutamine, proline, asparagine, L-glutathione reduced, betaine, taurine, choline, 2-aminobutyrate, tagatose, and 2-oxoglutarate and lower levels of L-alpha-phosphatidylinositol and D-sphingosine in the MHO group compared with MUHO. At the MUHNO group, significantly lower levels of alanine, glycine, glutamine, histidine, citrate, L-glutathione reduced, betaine and tagatose, and higher levels of isoleucine, L-proline, cholesterol, cholic acid, and carnitine were observed compared with MHNO individuals.

Correlation analysis of metabolites with HOMA-IR was performed in order to find the most relevant metabolite in obese and non-obese groups. In the obese group, D-sphingosine had a positive and choline had a negative correlation with the HOMA-IR. In non-obese people, we could not find any significant correlation (Tables 2, 3).

***Pathway enrichment analysis***

**Figure 4:**
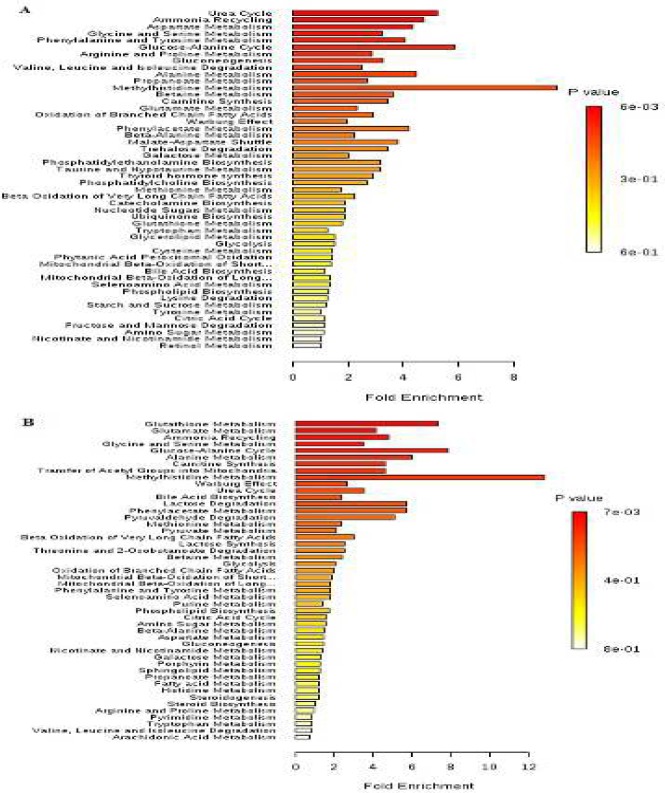
Pathway analysis overview showing altered metabolic pathways in comparison of (A) metabolically healthy obese (MHO) vs metabolically unhealthy obese (MUHO) and (B) metabolically healthy non-obese (MHNO) vs metabolically unhealthy non-obese (MUHNO) groups

Based on identified metabolites, pathway enrichment analysis was carried out using the MetaboAnalyst tool to reveal the most relevant pathways related to both groups (MHO vs MUHO) and (MHNO vs MUHNO). There were five altered pathways in the MUHO phenotype, including urea cycle, ammonia recycling, aspartate metabolism, glycine and serine metabolism, glucose-alanine cycle, and arginine and proline metabolism (Figure 4, Table 4). In parallel, five affected pathways were identified in the MUHNO individuals, including glutathione metabolism, glutamate metabolism, ammonia recycling, glycine and serine metabolism, glucose-alanine cycle, and alanine metabolism (Figure 4, Table 4).

## Discussion

Subgroups of obese (MHO) and non-obese (MUHNO) individuals revealed that cardiometabolic risk is not merely related to body weight (5). In this line, we used the untargeted metabolomics approach based on ^1^H-NMR combined with multivariate data analysis and discriminant modeling. We extracted metabolomic profiles of obese (MHO vs MUHO) and non-obese (MHNO vs MUHNO) groups and identified metabolites that distinguished metabolically healthy from unhealthy in each group. By comparing the metabolome of MHO vs MUHO individuals, several differential metabolites such as glutamine, asparagine, alanine, proline, L-glutathione reduced, 2-aminobutyrate, taurine, betaine, tagatose, choline, and D-sphingosine were uncovered. Differential metabolites in comparison of MHNO vs MUHNO individuals were alanine, glycine, glutamine, histidine, L-glutathione reduced, betaine, tagatose, isoleucine, L-proline, cholic acid, and carnitine. Moreover, as expected healthy status was associated with better metabolic profile including lower HOMA-IR index and insulin level and higher levels of HDL, irrespective of the presence or absence of obesity.

Metabolites are intermediates or products of various metabolic pathways, and thus shed light on metabolic flux through key biological pathways (23). Comparing the metabolome of MHO and MUHO subjects, several identified metabolites and related metabolism pathways were related to amino acid metabolism (aspartate metabolism, glycine and serine metabolism, glucose-alanine cycle, and arginine and proline metabolism), urea cycle, and ammonia recycling. Previous studies have emphasized the association of amino acid metabolism with obesity and T2DM, in which some increased or decreased amino acids in unhealthy phenotypes have been reported (18). It might be supposed that during impairment in amino acid metabolism along with urea cycle and ammonia recycling pathways in obesity, more ammonia has been produced and transported to the liver for excretion as urea in unhealthy condition. Ammonia can be also toxic to other organ systems. The results of one study showed that when intravenous ammonia was infused in dogs, hyperglycemia and increased plasma insulin concentrations occurred and it suggested that hyperammonemia induces insulin resistance (24). Our results also suggest that amino acid studying is efficient to improve the understanding of metabolic circumstances in the obesity phenotype. Glutamine level was increased in MHO subjects. Cheng *et al.* reported that glutamine is negatively correlated with obesity and IR in the experimental model. Indeed, glutamine improves cardiometabolic complications modulated by some possible mechanisms such as increased secretion of glucagon-like peptide 1, increased externalization of glucose transporter type 4, and/or increased adipose tissue insulin sensitivity (25). Asparagine level was also increased in the MHO phenotype. Two studies’ results showed asparagine is negatively correlated to unhealthy metabolic conditions (25, 26). Alanine is considered as regulator in glucose metabolism, and its level is increased in MHO individuals. The results of Brennan *et al.* study demonstrated that L-alanine metabolism, in addition to the enhancing effect on glucose metabolism, contributes to the stimulatory effects of this amino acid on improvement of insulin secretion *in vitro* (27). Besides, three antioxidant metabolites, L-glutathione reduced (GSH), 2-aminobutyrate, and taurine had higher levels in MHO individuals. Hepatic antioxidant glutathione (GSH) homeostasis has a pivotal role in cellular defense against oxidative stress (28). 2-Aminobutyrate increases intracellular glutathione levels by activating AMPK, which has a key function in NADPH maintenance and protects against oxidative stress (29). Taurine provides sufficient pH buffering in the mitochondrial matrix by which it indirectly acts as an antioxidant. Therefore, taurine prohibits the obesity progression by maintaining mitochondrial function (30). In addition, choline and betaine levels were higher in MHO individuals. Choline had negative correlation with HOMA-IR in our analysis. Choline is an immediate metabolic precursor of betaine, and they are metabolically related quaternary ammonium compounds. The results of one study showed that higher intake of dietary choline and betaine is associated with lower IR in the general population (31). Betaine supplementation ameliorates extracellular signal regulated kinases 1/2 and protein kinase B activations by which it enhances insulin sensitivity in adipose tissue (32). Tagatose level was higher in MHO subjects. Tagatose supplement as an epimer of fructose significantly improved weight loss and HDL level in diabetes patients’ diet (33). Tagatose is a drug which now is under development for the treatment of diabetes and to control obesity (34). Finally, D-Sphingosine had lower level in MHO individuals, and it positively correlated with HOMA-IR in our study. The results of one study showed that sphingosines were found to be significantly higher in adipose tissues of metabolically unhealthy versus healthy obese (35). Sphingosine is the byproduct of ceramide. Ceramide and sphingosine can prohibit insulin signaling by inhibiting Akt and AMPK in *in vitro* studies (36).

The second part of our results was related to the comparison of metabolically healthy versus unhealthy non-obese individuals. It is noticeable that amino acid metabolism (glutamate metabolism, glycine and serine metabolism, glucose-alanine cycle, and alanine metabolism) and ammonia recycling were the major affected pathways in MUHNO individuals, like in the MUHO phenotype. As earlier discussed, ammonia recycling and impaired amino acid metabolism might result in hyperammonemia, which induces insulin resistance (24). Glutathione metabolism was another altered pathway in MUHNO individuals. Glutathione is one of the major components of the antioxidant defense system. Numerous studies demonstrated the association of increased oxidative stress and insulin resistance pathogenesis mediated by insulin signal inhibition and adipokines dysregulation (37). Our results also suggested the possible roles of amino acids in metabolic circumstances existing in non-obese phenotype. Glycine level was increased in MHNO individuals. Glycine influences glucose metabolism. It could induce insulin secretion through glucagon-like peptide-1 and glucose-dependent insulinotropic polypeptide (38). An earlier study reported that lean non-diabetic insulin-resistant offspring of diabetic parents who showed several grades of defects in their insulin secretion represented low plasma glucogenic amino acid concentrations, specifically glycine levels (39). Isoleucine is a branched-chain amino acid (BCAA), which had lower level in MHNO individuals. Recent studies have shown that the elevated serum BCAAs is associated with metabolic disorders, which is not dependent on body weight as higher levels have been found in metabolically unwell individuals (40). The results of two studies showed that the increased level of isoleucine was positively correlated to IR values (18, 26). Moreover, cholesterol and cholic acid had lower levels in MHNO individuals. Bile acid synthesis is the major pathway for catabolism of cholesterol to bile acids. The results of one study showed that plasma cholic acid was negatively associated with insulin sensitivity in a variety of subjects, including healthy volunteers, and patients with obesity and T2DM. It was worthwhile to note that HOMA-IR remained positively related to those plasma bile acid levels in multivariable analysis (41). At the last, carnitine level was lower in MHNO individuals. Carnitine transfers fatty acids into the mitochondrial matrix for fatty acid oxidation. Current studies suggest that overexposure of lean-tissues to fatty acid may lead to mitochondrial fatty acid overload and surplus oxidation. This situation results in increased radical oxidative stress and induced insulin resistance (42).

Our metabolomics study presented a holistic view of metabolic changes related to metabolically healthy *vs* unhealthy in obese and non-obese individuals. As shown in a prospective population-based study (43), either metabolically healthy obese or metabolically unhealthy non-obese individuals are exposed at increased risk of cardiovascular disease and diabetes. In this line, it is worth emphasizing that the median age of our study participants was 23–35 in obese and 24–30 years in non-obese, which may help to predict the risk of cardiometabolic disorders, and it is more important about metabolically unhealthy non-obese individuals who appear to be at lower risk of cardiometabolic events due to their body weight. However, our study has limitations including a relatively small sample size in each group, which might fail to detect some differential metabolites. Besides, it should be better regarding diet and physical activity of all subjects, but it was not possible for us. Finally, there is a need to make sequential observations in a prospective manner to provide more useful information.

**Table 1:** Baseline characteristics of obese and non-obese phenotypes

	MHO	MUHO	*P-*value	MHNO	MUHNO	*P-*value
Age(yr)	29 (25.0-35.0)	26 (23-33)	0.2	25 (24-29.25)	26 (24.5-30)	0.4
Gender (%) Male Female	38.1 61.9	47.6 52.4	0.5	59.1 40.9	54.5 45.5	0.7
BMI (kg/m^2^)	31.7 (30.45-32.60)	31.2 (30.5-33.3)	0.4	23.15 (21.97-25.5)	24.75 (22.15-26.27)	0.9
SBP (mmHg)	100 (100-110)	110 (100-115)	0.3	110 (97.5-112.5)	110 (100-120)	0.3
DBP (mmHg)	70 (70-80)	70 (70-80)	0.2	70 (70-80)	70 (60-80)	0.4
FBS (mg/dl)	88 (83-95.5)	86 (82-92)	0.6	84.5 (80-88)	85 (80-89.25)	0.6
Total cholesterol (mg/dl)	181 (156-199)	172 (157.5-198)	0.2	176 (167.5-214)	157.5 (140.75-173.75)	0.003
TG (mg/dl)	97 (87.5-121)	105 (88.5-136.5)	0.4	86 (80.5-104.0)	97.5 (71.0-121.5)	0.4
HDL (mg/dl)	46 (44.0-50.5)	35 (32-38)	0.00	45.5 (42.0-53.5)	35.5 (33.0-39.0)	0.00
Insulin (µIU/L)	11.48 (9.28-13.75)	14.26 (10.66-24.81)	0.04	7.78 (6.0 -11.31)	9.93 (8.41-12.66)	0.01
HOMA-IR	2.49 (1.85-2.98)	2.92 (2.17-5.26)	0.04	1.6 (1.18-2.40)	2.11 (1.73-2.9)	0.02
HOMA-IR (%)	47.6%	57.1%	0.5	22.7%	27.3%	0.7

Data are presented as median and interquartile range or in percentage.

metabolically healthy obese (MHO), metabolically unhealthy obese (MUHO), metabolically healthy non-obese (MHNO), metabolically unhealthy non-obese (MUHNO), BMI; body mass index, SBP; systolic blood pressure, DBP; diastolic blood pressure, FBS; fasting blood sugar, TG; triglyceride, HDL; high density lipoprotein, HOMA-IR; Homeostatic Model Assessment-Insulin Resistance

**Table 2:** Number of important serum metabolites and their correlation with insulin resistance in metabolically healthy obese (MHO) individuals compared with metabolically unhealthy obese (MUHO) ones

Metabolites	d ^1^H (p.p.m.)^*^	VIP^**^	Fold change ^**^	*P-*value	Direction of variation^****^	Correlation with HOMA-IR	*P-*value
Alanine	1.47	1.01	1.88	0.033	+	-0.18	0.38
Glutamine	3.77	1.50	1.55	0.000	+	-0.19	0.38
Proline	2.33	1.03	1.67	0.01	+	-0.34	0.10
Asparagine	6.91	1.79	1.69	0.000	+	-0.3	0.11
L-Glutathione reduced	3.76	1.51	1.55	0.021	+	-0.19	0.38
Betaine	3.25	1.01	1.88	0.01	+	-0.28	0.9
Taurine	3.41	0.62	1.59	0.000	+	-0.22	0.29
Choline	3.19	1.53	1.51	0.000	+	-0.5	0.01^*^
2-Aminobutyrate	0.97	1.64	1.69	0.000	+	0.26	0.21
Tagatose	3.73	1.49	1.51	0.000	+	0.2	0.3
2-Oxoglutarate	2.43	1.66	2.13	0.036	+	0.3	0.21
L-alpha-phosphatidylinositol	1.31	1.02	1.63	0.006	-	0.3	0.08
D-Sphingosine	1.25	1.25	3.92	0.012	-	0.44	0.03^*^

* Chemical shift of signal was used for quantification

** Variable influence on projection (VIP) was used to rank variables

*** Fold change >1.5 was considered as significant

**** Plus and minus marks mean increase and decrease in MHO compared with MUHO

**Table 3:** Number of important serum metabolites and their correlation with insulin resistance in metabolically healthy non-obese (MHNO) individuals compared with metabolically unhealthy non-obese (MUHNO) ones

Metabolites	d 1H (p.p.m.)^*^	VIP^**^	Fold change^***^	*P-*value	Direction of variation^****^	Correlation with HOMA-IR	*P-*value
Alanine	1.47	2.19	7.41	0.01	+	-0.3	0.30
Glycine	3.55	1.35	2.93	0.000	+	-0.1	0.60
Glutamine	3.77	2.13	2.41	0.000	+	-0.5	0.10
Histidine	7.73	0.88	1.70	0.001	+	-0.2	0.15
Citrate	2.53	1.98	3.19	0.000	+	-0.5	0.07
L-Glutathione reduced	2.15	1.15	1.8	0.000	+	-0.1	0.7
Betaine	3.25	2.00	4.07	0.03	+	-0.4	0.1
Tagatose	3.73	1.88	1.86	0.011	+	-0.01	0.9
Isoleucine	1.97	1.19	2.27	0.000	-	0.3	0.2
L-Proline	3.37	1.98	1.95	0.000	-	0.2	0.3
Cholesterol	0.83	1.30	1.51	0.001	-	0.4	0.2
Cholic acid	3.17	2.04	3.39	0.02	-	0.3	0.2
Carnitine	3.43	1.94	1.78	0.000	-	0.3	0.2

* Chemical shift of signal was used for quantification

** Variable influence on projection (VIP) was used to rank variables

*** Fold change >1.5 was considered significant

**** Plus and minus marks mean increase and decrease in MUHNO compared with MHNO

**Table 4:** Significant enriched pathways related to comparison of metabolically healthy (MHO) vs unhealthy (MUHO) obese and metabolically healthy (MHNO) vs unhealthy (MUHNO) non-obese

Pathway	Enriched metabolites	*P*-value
**Pathway (MHO ** ***vs*** ** MUHO)**		
Urea cycle	Alanine, Oxoglutaric acid, Arginine, Glutamine	0.00581
Ammonia recycling	Asparagine, Histidine, Oxoglutaric acid, Glutamine	0.00832
Aspartate metabolism	Asparagine, Oxoglutaric acid, Arginine, Glutamine	0.0115
Glycine and Serine metabolism	Betaine, Creatine, Alanine, Oxoglutaric acid, Arginine	0.0162
Glucose-Alanine cycle	Alanine, Oxoglutaric acid	0.0437
Arginine and Proline metabolism	Creatine, Proline, Oxoglutaric acid, Arginine	0.0463
**Pathway (MHNO ** ***vs*** ** MUHNO)**		
Glutathione metabolism	Glycine, Glutathione, Alanine	0.00678
Glutamate metabolism	Glutamine ,Glycine, Glutathione, Alanine	0.0127
Ammonia recycling	Glycine, Histidine, Glutamine	0.022
Glycine and serine metabolism	Betaine, Glycine, Alanine, Threonine	0.0241
Glucose-alanine Cycle	D-Glucose, Alanine	0.0249
Alanine metabolism	Glycine, Alanine	0.0414

## Conclusion

We identified differential metabolites that could distinguish obese (MHO vs MUHO) and non-obese (MHNO vs MUHNO) individuals. Literature review helped us to point that the increased or decreased levels of the most differential metabolites might improve insulin sensitivity in MHO individuals, while they might contribute to insulin resistance in MUHNO ones. Furthermore, it may have merit to highlight that amino acids as well as impairment in their metabolism and ammonia recycling pathways might be related to insulin resistance in the metabolically unhealthy phenotypes, irrespective of the presence or absence of obesity. Thus, future studies are required to investigate the relation of identified metabolites with the impairment of relevant metabolic pathways in metabolically unhealthy conditions in obese and non-obese individuals.
